# Public Clonotypes and Convergent Recombination Characterize the Naïve CD8^+^ T-Cell Receptor Repertoire of Extremely Preterm Neonates

**DOI:** 10.3389/fimmu.2017.01859

**Published:** 2017-12-19

**Authors:** Alison J. Carey, Jennifer L. Hope, Yvonne M. Mueller, Adam J. Fike, Ogan K. Kumova, David B. H. van Zessen, Eric A. P. Steegers, Mirjam van der Burg, Peter D. Katsikis

**Affiliations:** ^1^Department of Pediatrics, Drexel University College of Medicine, Philadelphia, PA, United States; ^2^Department of Microbiology and Immunology, Drexel University College of Medicine, Philadelphia, PA, United States; ^3^Department of Immunology, Erasmus University Medical Center, Rotterdam, Netherlands; ^4^Department of Bioinformatics, Erasmus University Medical Center, Rotterdam, Netherlands; ^5^Department of Obstetrics and Gynecology, Erasmus University Medical Center, Rotterdam, Netherlands

**Keywords:** preterm, neonate, T-cell receptor repertoire, convergent recombination, public clonotypes, diversity analysis

## Abstract

Respiratory support improvements have aided survival of premature neonates, but infection susceptibility remains a predominant problem. We previously reported that neonatal mice have a rapidly evolving T-cell receptor (TCR) repertoire that impairs CD8^+^ T cell immunity. To understand the impact of prematurity on the human CD8^+^ TCR repertoire, we performed next-generation sequencing of the complementarity-determining region 3 (CDR3) from the rearranged TCR variable beta (Vβ) in sorted, naïve CD8^+^ T cells from extremely preterm neonates (23–27 weeks gestation), term neonates (37–41 weeks gestation), children (16–56 months), and adults (25–50 years old). Strikingly, preterm neonates had an increased frequency of public clonotypes shared between unrelated individuals. Public clonotypes identified in preterm infants were encoded by germline gene sequences, and some of these clonotypes persisted into adulthood. The preterm neonatal naïve CD8^+^ TCR repertoire exhibited convergent recombination, characterized by different nucleotide sequences encoding the same amino acid CDR3 sequence. As determined by Pielou’s evenness and iChao1 metrics, extremely preterm neonates have less clonality, and a much lower bound for the number of unique TCR within an individual preterm neonate, which indicates a less rich and diverse repertoire, as compared to term neonates, children, and adults. This suggests that T cell selection in the preterm neonate may be less stringent or different. Our analysis is the first to compare the TCR repertoire of naïve CD8^+^ T cells between viable preterm neonates and term neonates. We find preterm neonates have a repertoire immaturity which potentially contributes to their increased infection susceptibility. A developmentally regulated, evenly distributed repertoire in preterm neonates may lead to the inclusion of public TCR CDR3β sequences that overlap between unrelated individuals in the preterm repertoire.

## Summary

Analysis of the naïve CD8^+^ TCR repertoire of human preterm neonates, term neonates, children and adults was performed, which revealed reduced diversity and richness in preterm neonates. Repertoire differences in the extremely preterm neonate may have implications for their immune competence.

## Introduction

Approximately one in four extremely premature infants born between 22 and 28 weeks of gestation does not survive the birth hospitalization (1). Despite a reduction in death rates attributed to respiratory distress syndrome and bronchopulmonary dysplasia in extremely premature infants over the past 20 years (2), death caused by infection has remained the same or has increased in rate (2, 3). Specifically, respiratory viral infections contribute substantially to global fetal and infant losses and disproportionately affect preterm neonates. Of all deaths secondary to a respiratory viral infection, 55% occur in a neonate born before 30 weeks gestation (4). Cytotoxic CD8^+^ T lymphocytes (CTL) are critical for protection from viruses and intracellular bacteria (5, 6). The CTL responses in neonates are less robust (7, 8), which leads to reduced protection in this sensitive age group. Prematurity may further impair CTL responses and increase susceptibility to infections.

In the context of a respiratory infection such as influenza virus, the neonatal mouse CTL response is delayed compared to adult or adolescent mice (9, 10). The expansion of viral-specific CTLs is significantly slower and their TCR affinity is lower, and this correlates with an increased rate of morbidity and mortality. A contributing mechanism to this altered CD8^+^ T cell response is a more oligoclonal naïve T-cell receptor (TCR) repertoire, which rapidly becomes more diverse from 3 to 7 days postpartum (9). This more restricted neonatal repertoire is in contrast to the adult mouse’s diversified TCR repertoire (9, 11). This has a significant impact on the ability of the adaptive immune system to specifically recognize pathogens and mount protective responses, as this largely depends on the expression of a highly diversified set of TCRs.

The above findings in mice raised the question whether such rapid evolution of the TCR repertoire also occurs between preterm and term human neonates, which would result in qualitative differences in CTL-mediated protection in preterm neonates. Generation of a functional immune repertoire is a developmental process that initiates during fetal life but matures only several years after birth in humans. T cell progenitors begin migrating to the thymus by 8–9 weeks of gestation and circulating mature T lymphocytes are detected at 15–16 weeks of gestation (12). Previous studies using PCR amplification have indicated that the TCR complementarity-determining region 3 (CDR3) length is shorter during human fetal life and that this length gradually increases in size until term (13, 14). In term neonates, there is evidence of lower numbers of added nucleotides within the CDR3 region and relatively high numbers of clonotypes shared, which indicates a germline repertoire (15, 16). In addition, the IgG repertoire of extremely preterm neonates is more germline gene encoded, and only has an increase in somatic mutation frequency when they pass a post-conceptual age corresponding to term gestation (17).

However, there has been little investigation of the viable, extremely preterm neonate’s TCR repertoire, particularly using next-generation sequencing (NGS) technologies that allow an unprecedented depth of sampling. Recently, Rechavi and colleagues looked at the total T cell repertoire by NGS in a small number of fetal samples from selective fetal reduction cases, 12–26 weeks gestation, compared to children aged 9–48 months (18). Although these studies revealed differences between total T cell fetal TCR repertoire and children, they do not address the contribution or evolution of the CD8^+^ T cell TCR repertoire between neonates and children. Importantly, the question remains whether viable premature neonates and term neonates differ in their TCR repertoire and whether such differences would impact immunity in premature neonates.

Given the susceptibility of preterm infants to intracellular pathogens, such as respiratory viruses (19), and the importance of CD8^+^ T cells in combating these pathogens, we sought to determine by NGS the TCR repertoire of naïve CD8^+^ T cells in viable, live-born extremely preterm neonates (23–27 weeks gestation) and compared them to term neonates (37–41 weeks gestation), young children (16–56 months), and adults (25–50 years old). We report that preterm neonates have a less rich naïve CD8^+^ TCR repertoire, characterized by public TCR sequences which are closer to germline and exhibit convergent recombination. The pattern of sharing decreases steadily over time.

Such repertoire differences from term neonates could contribute to the increased susceptibility of preterm neonates to infections during their first months of life. To our knowledge, this is the first report of the comparison of naïve CD8^+^ TCR repertoire development across the human life span, from the edges of neonatal viability to adulthood.

## Materials and Methods

### Cord and Peripheral Blood Collection

Cord blood sampling kits were preassembled to standardize collection procedures, ensuring uniform reagents were used (20). Mixed venous arterial cord blood was collected from five elective, full-term cesarean section deliveries (37–41 weeks gestation), with no rupture of membranes or labor and from four extremely preterm deliveries (23–27 weeks gestation). Detailed clinical information about the four preterm samples is in Table S1 in Supplementary Material. Peripheral venous blood was also obtained from five healthy children ages 16–56 months of age, who were undergoing elective, routine surgery, and from five control healthy, nonsmoking adults (aged 20–50 years; median age 39 years). Study data were collected and managed using REDCap electronic data capture tools hosted at Drexel University (21). REDCap (Research Electronic Data Capture) is a secure, web-based application designed to support data capture for research studies. All specimens and data were de-identified. Cord blood was collected and stored at room temperature until processing within 4 h of collection at the Drexel University College of Medicine, Department of Microbiology and Immunology or Erasmus Medical Center, Department of Immunology.

### Cord and Peripheral Blood Mononuclear Cell Isolation

All processing was completed using BSL 2 procedures. Prior to cell isolation, a blood aliquot was diluted with one part 10% RPMI to one part whole blood in a sterile 50 mL conical tube. Diluted blood was layered over Ficoll-Paque^®^ (Lymphocyte Separation Medium, Cellgro) at a ratio of 3 mL blood to 1 mL separation medium. Blood was pipetted slowly down the side of the 50 mL conical to overlay the Ficoll-Paque. Samples were centrifuged at 20°C for 30 min at 900 *g* with no brake. After centrifugation the mononuclear cell layer was removed and transferred to a new 50 mL conical. The tube was filled with 5% RPMI and centrifuged for 5 min at 500 *g* at 20°C, with the brake. Cells were washed with 5% RPMI, and then resuspended in 10% RPMI. Cells were frozen in 1 × 10^7^ cells/mL aliquots in freeze media, 10% DMSO (Sigma) and 90% sterile-filtered prescreened FBS (Gemini Bioproducts). Cells were kept in long-term storage in −150° freezers.

### Cell Sorting and TCR Sequencing

For the sorting of naïve CD8^+^ T cells, cells were thawed and stained with Annexin V-Cy5.5, anti-CD3-BV421, anti-CD8-BV786, anti-CCR7-PE-CF594, and anti-CD45RA-APC-H7 for 30 min on ice, washed once with HBSS/3% FBS/2.5 mM CaCl_2_, and resuspended in HBSS/3% FBS/2.5 mM CaCl_2_. All antibodies were purchased from BD Bioscience (San Diego, CA, USA). Live (Annexin V negative), naïve CD8^+^ T cells (CD3^+^CD8^+^CD45RA^+^CCR7^+^) were sorted on a FACSAria III cell sorter (BD Biosciences) and the cell pellet was snap-frozen. gDNA extraction was performed following the DNeasy Blood and Tissue Kit (Qiagen). Sorted cell numbers ranged from ~20,000 to ~100,000. Sample data were generated using the immunoSEQ^®^ Assay (Adaptive Biotechnologies, Seattle, WA, USA). The somatically rearranged TCR CDR3 was amplified from genomic DNA using a two-step, amplification bias-controlled multiplex PCR approach. Primers specific for every V and J gene segment amplified the hypervariable CDR3 of the TCR beta locus and CDR3 libraries were sequenced.

### Naïve CD8^+^ TCR Repertoire Analysis

Sequencing data were filtered and clustered using both the relative frequency ratio between similar clones and a modified nearest-neighbor algorithm, to merge closely related sequences. CDR3 segments were annotated according to the International ImMunoGeneTics (IMGT) collaboration, identifying which V, D, and J genes contributed to each rearrangement (www.imgt.org). TCR CDR3 sequences were then normalized to correct for residual multiplex PCR amplification bias and quantified against a set of synthetic TCR CDR3 sequence analogs. CDR3 sequences were organized providing in-frame and out-of-frame sequences. An algorithm was applied to the in-frame sequences for collapsing reads, which resulted in unique in-frame rearrangements of the CDR3 genes. Sequences were classified as nonproductive if it was determined that non-templated insertions or deletions produced frameshifts or premature stop codons. In-frame unique sequences without stop codons are referred to as unique productive sequences and are the object of this study. Data were analyzed using the immunoSEQ Analyzer toolset. Circos plots were generated with the Circos software package (22). The sequencing data for the 19 individual samples used for naive repertoire analysis can be accessed from https://clients.adaptivebiotech.com/pub/carey-2017-frontiersinimmunology and https://doi.org/10.21417/B7X334.

### Diversity Metric Calculation

The Clonality index and iChao1, a lower bound on repertoire richness, were calculated using the Adaptive Biotechnologies Immunoseq Analyzer software. The Clonality index is defined as 1-Pielou’s Evenness, a normalized Shannon’s H entropy (23). The Clonality index and Pielou’s Evenness measure the repertoire distribution. For the Clonality index, values approaching 1 indicate a very skewed distribution of frequencies, and values approaching 0 indicate that every rearrangement is present at nearly identical frequency. iChao1 is a non-parametric estimator of the lower bound of the total number of unique templates within an individual’s repertoire (24). The lower bound is the minimum number of unique templates predicted to be within an individual’s repertoire, with a 95% confidence interval.

### Statistical Analysis

For continuous variables, the Shapiro–Wilk *W* test for normality was systematically performed for each group. When parametric conditions were fulfilled, an unpaired Student’s *t*-test was performed. For non-parametric conditions, a Wilcoxon signed-rank test for unpaired samples was performed. Analyses were performed with the JMP statistical analysis program (SAS, Cary, NC, USA). The tests used are indicated in figure and table legends. A *p* value below 0.05 was considered statistically significant.

### Study Approval

This study was approved by the Institutional Review Board of Drexel University College of Medicine and Erasmus Medical Center. Those patients with fetal demise, maternal active HSV infection, and mothers with known HIV infection were excluded. There was a waiver of documentation of informed consent for cord blood collection because of the minimal risk. For peripheral blood collection from children and adults, written informed consent was obtained. Subjects gave written informed consent in accordance with the Declaration of Helsinki.

## Results

### Human TCR Vβ Family Usage Matures by the End of the Second Trimester of Pregnancy

**Figure 1:**
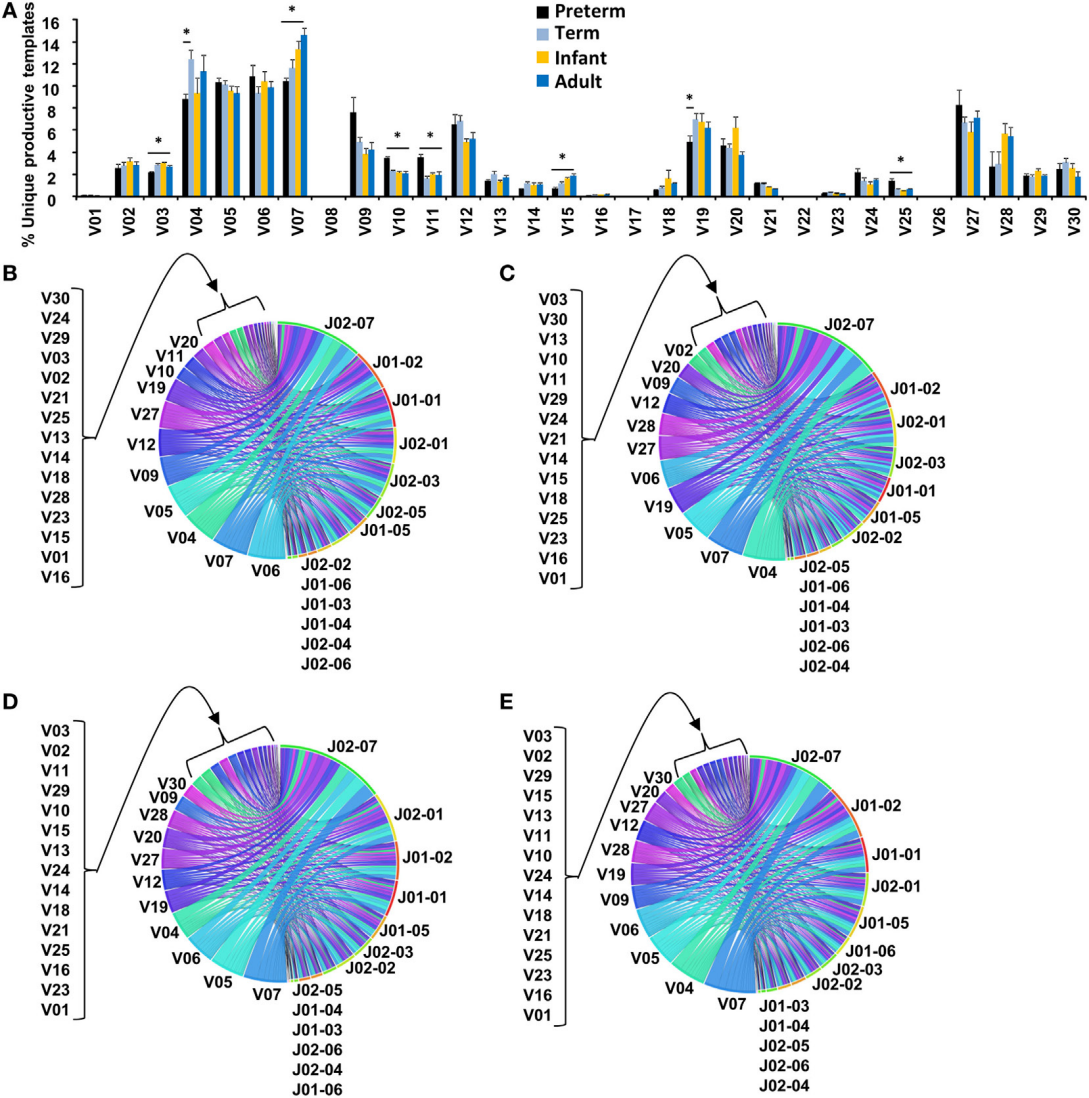
VJ joining in the human repertoire is mature by the end of the second trimester of pregnancy. **(A)** The percentage of total Vβ gene family usage (mean ± SEM) of all unique productive templates shown. Unpaired Student’s *t*-test was performed to compare an age group to one other age group. * represents *p* < 0.05. Representative Circos plots of frequencies of Vβ and Jβ usage and combinations of unique productive templates shown for **(B)** preterm neonate, **(C)** term neonate, **(D)** young child, and **(E)** adult. The width of the band is proportional to the frequency.

To investigate the human naïve CD8^+^ TCR repertoire, we performed high-throughput sequencing of the TCRβ CDR3 regions in FACS-sorted, naive CD8^+^ T cells from extremely preterm neonates (23–27 weeks gestation, *n* = 4), term neonates (37–41 weeks gestation, *n* = 5), young children (16–56 months, *n* = 5), and adults (25–50 years, *n* = 5) (Table S2 in Supplementary Material). Although preterm neonates had variation in the total number of productive templates, overall there was no difference among the four groups in terms of average templates (Figure S1 in Supplementary Material). To initially assess diversity of the T cell repertoire, TCRβ variable (TRBV) and TCRβ joining (TRBJ) combinations in the total repertoire were analyzed in each age group. Others have previously reported a skewed usage of TRBV families early in fetal development (18), and we questioned whether the extremely preterm neonate would have different TRBV usage as compared to their term counterparts. There were some statistically significant differences in TRBV usage between preterm neonates and the older age groups (Figure 1A), although no clear skew. TRBV04, TRBV05, TRBV06, TRBV07, and TRBV27 were the most common families used and collectively made up ~50% of the unique productive repertoire in all age groups examined, indicating that the stochastic rearrangement in the thymus is similar in these groups. Circos plots of V-J recombination usage were generated for the total unique repertoire in each age group (Figures 1B–E) and revealed no biased usage of TRBV and TRBJ. Thus, the extremely preterm neonate has TRBV usage which is well-diversified by the 27th week of gestation or the end of the second trimester.

### The Most Frequent Clones in the Extremely Preterm Neonate Exhibit Differential TCR Vβ Family Usage

**Figure 2:**
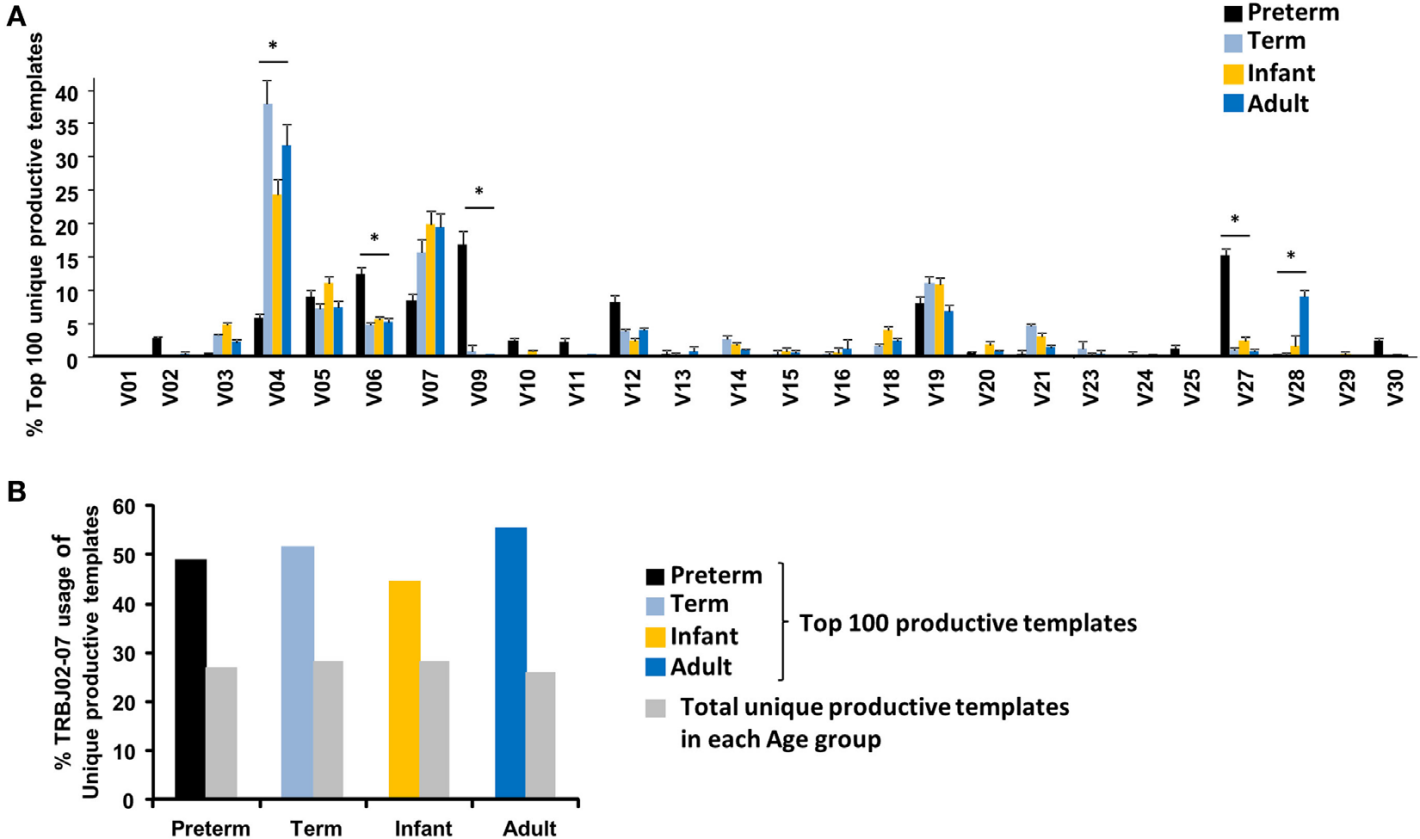
Differential TCR Vβ family usage in the extremely preterm neonate’s most frequent clones. **(A)** The frequency of Vβ gene family usage (mean ± SEM) for the top 100 unique productive templates within each age group is shown. Unpaired Student’s *t*-test was performed to compare an age group to one other age group. * represents <0.05. **(B)** The frequency of TRBJ02–07 usage in the total unique *versus* the top 100 repertoire (mean).

We next looked at TRBV gene usage and VJ combinations of the 100 most frequent clones in each age group to uncover any bias. We found an enrichment in the top 100 clones in TRBV04 usage in all ages except the preterm neonates. This enrichment was already present in term neonates and TRBV04 increased from 12.4% in overall repertoire (Figure 1A) to 37.9% in top 100 clones (Figure 2A). In contrast, preterm neonates TRBV04 usage dropped from 8.9% in the overall repertoire to 6.0% in the top 100 clones. Preterm usage of TRBV09 increased from 7.6% in overall repertoire to 17.0% in the top 100 clones, while in the other three groups it decreased from an average of 4% to less than 1% (Figures 1A and 2A). TRBV27 usage differed only slightly in preterm neonates between the top 100 clones and the overall repertoire (15.5 and 8.3%, respectively; Figures 1A and 2A), but its usage was decreased in the top 100 clones in all other 3 age groups (1.5 and 6.5% in top 100 clones and the overall repertoire, respectively; Figures 1A and 2A). In the most prevalent clones, there is a skew in the older samples toward using the TRBV04, TRBV07, and TRBV19 families which make up ~60% of the top 100 clones, while in preterm neonates, TRBV05, TRBV06, TRBV09, and TRBV27 constitute 50% of the top 100 clones (Figures 1A and 2A). Although there is fairly equal distribution of J gene usage in the total repertoire (Figures 1B–E), the most prevalent clones are dominated by TRBJ02–07 in all age groups, with TRBJ02–07 being present in approximately half of the 100 most common clones (Figure 2B). Thus, the TRBV family usage in the overall naïve CD8^+^ TCR repertoire is similar in all age groups, but the preterm neonates do use very different TRBV families for their most common clones.

### TCR of Preterm Neonates Are Closer to Germline

**Figure 3:**
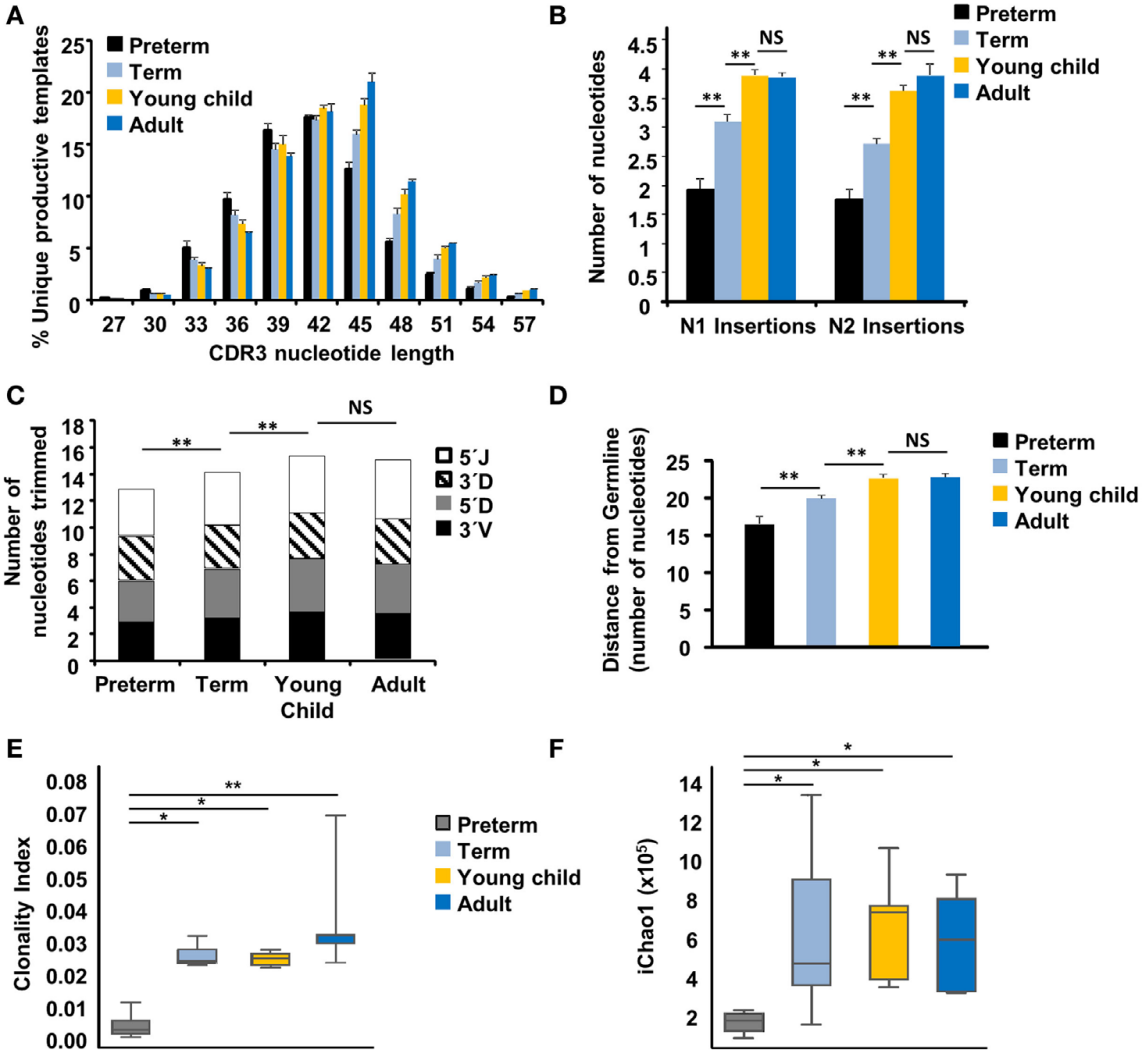
The TCR repertoire of naïve CD8^+^ T cells from preterm neonates is close to germline. **(A)** The complementarity-determining region 3 (CDR3) sequence frequency (mean ± SEM) for all unique productive templates for the extremely preterm neonates, term neonates, young children and adults is shown. **(B)** The number of nucleotides (mean ± SEM) inserted into the N1 (region between the V and J gene segments) and N2 (region between the J and D gene segments) are shown. **(C)** 3′V and 3′D trimming is the nucleotide loss at the 3′of the V and D gene segments, respectively; 5′J and 5′D trimming is the nucleotide loss at the 5′ of the J and D gene segments, respectively. **(D)** Distance from germline is calculated based on the average number of nucleotides ± SEM inserted or trimmed at the gene junctions. The Clonality index (1 − Pielou’s Evenness) **(E)** and the richness as determined by iChao1 **(F)** of the naïve repertoire for all four age groups. Shapiro–Wilk test to confirm normal distribution and an unpaired Student’s *t*-test was performed. * represents <0.05; ** represents *p* < 0.01.

The average weighted CDR3 length of preterm neonates was 41.3 nucleotides, as compared to 42.4 nucleotides for term neonates (*p* < 0.05), 43.0 for young children (*p* < 0.01), and 43.4 for adults (*p* < 0.01). In addition to preterm neonates having shorter average weighted CDR3 lengths as compared to other age groups, the distribution of CDR3 lengths is different (Figure 3A), with preterm neonates having fewer sequences with longer lengths of 45 or 48 base pairs. Deconstruction analysis of the CDR3 region showed that the reduced length of the CDR3 lengths in neonatal samples was due to reduced N nucleotide addition (Figure 3B), as previously reported (18, 25). There was a significant difference (*p* < 0.01) in the number of N1 and N2 additions between the preterm and term neonates, and between the term neonates and young children, indicating a stepwise progression from the *in utero* environment over the first few years of life. In addition, there was decreased trimming at the 5′ and 3′ ends of the *VDJ* genes (Figure 3C), indicating that reduced exonucleolytic activity contributes to the generation of more germline CDR3 regions in preterm neonates. Distance from germline was calculated by taking the absolute number of nucleotides added in the N1 and N2 regions and removed from the *VDJ* gene junctions. This revealed that the preterm repertoire was closer to germline as compared to their older counterparts (Figure 3D). Taken together, the preterm TCR had a shorter CDR3 region, with fewer N additions and junctional trimming and was closer to germline compared to term neonates, children, and adults.

### The Naïve CD8^+^ TCR Repertoire Is Evenly Distributed and Less Rich in Preterm Neonates

We next sought to determine differences in repertoire diversity across the life span. Diversity has two independent components: clonality and richness. To address clonality, we calculated the Clonality index, defined as 1 − Pielou’s evenness, to determine differences in clonality between the age groups. A high clonality index score approaching 1 indicates oligoclonality, whereas a low clonality score approaching 0 indicates there is very little clonal expansion, without a particular clone dominating the repertoire. Preterm neonates have a very even distribution of their repertoire, and therefore have a statistically significant lower clonality, as compared to their term, young child, and adult counterparts (*p* < 0.02) (Figure 3E). In general, the sorted, naïve CD8^+^ T cells for all individuals had little clonal expansion, as expected. However, the maintenance of the human naive T cell pool occurs almost exclusively through peripheral T cell division (26), and therefore, this finding likely represents differences in homeostatic proliferation in the preterm neonate.

Next, we sought to determine the richness of the individual repertoires. Richness is defined as the number of distinct species or unique TCR in a sample or population. To estimate richness in our TCR repertoire, we used the iChao1 index that utilizes TCR templates seen once or twice to estimate the lower bound of richness of TCR species within a repertoire (27). The iChao1 index was significantly lower in the preterm neonates, compared to all other age groups (*p* < 0.05) (Figure 3F). Taken together, preterm neonates have an even distribution of clones, but also have a much lower bound for the total number of unique templates within an individual preterm neonate, which indicates a less rich repertoire. The above demonstrate that although TRBV usage is stochastic and similar in all age groups, preterm neonates release into the periphery an even and far less rich TCR repertoire than term neonates, children, and adults; this suggests that T cell selection in the preterm neonates may be less stringent or different.

### Public Clones Are Enriched in the Preterm Naïve CD8^+^ TCR Repertoire

**Figure 4:**
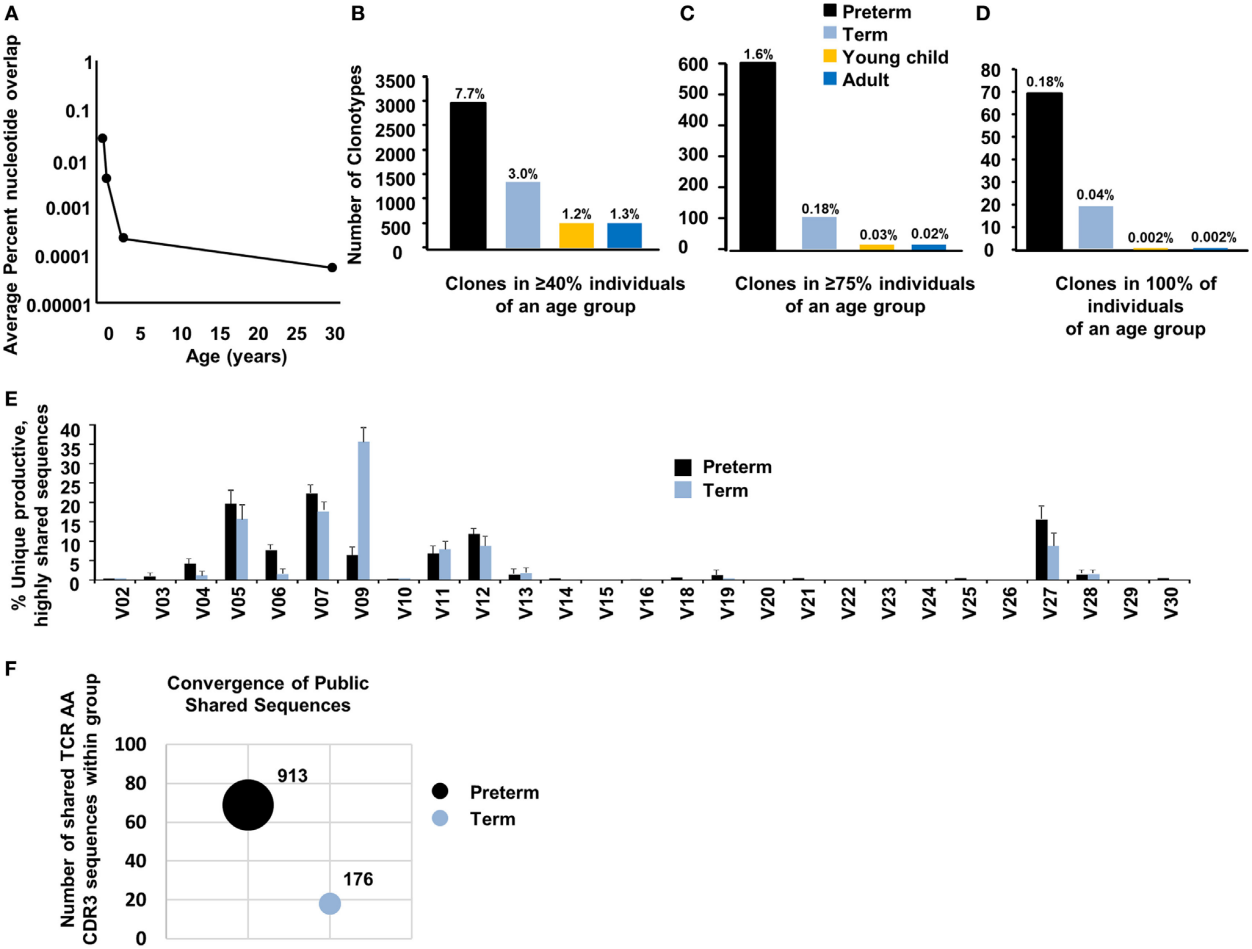
Public clones are enriched in the preterm TCR repertoire. **(A)** Average percent nucleotide overlap within each age group. The number of clonotypes which are shared with **(B)** at least 40%, **(C)** at least 75%, and **(D)** 100% of the individuals within that particular age group is depicted. **(E)** The percentage of Vβ gene family usage (mean ± SEM) for the highly public (shared in greater than or equal to four of the individuals) unique productive sequences within each preterm and term neonate is shown. **(F)** Bubble plot comparing TCR convergence between preterm and term neonate highly shared TCR present in all individuals. Bubble size relative to the number of single TCR nucleotide sequences contributing to shared complementarity-determining region 3 (CDR3) sequences.

Based on the less rich TCR repertoire in preterm neonates, we next sought to quantitate the number of public sequences within each age group. A developmentally regulated, even, less stringently selected repertoire in preterm neonates may lead to the inclusion of public TCR CDR3 sequences that overlap between unrelated individuals in the preterm repertoire. We therefore questioned whether there were common TCR nucleotide sequences among the four preterm neonatal samples and whether sharing of common TCR nucleotide sequences would be similar in the other age groups. We found that preterm neonates had an overall average increased sharing at the nucleotide level, as compared to their older counterparts (Figure 4A). Preterm neonates had 2.7% (1338/49622 nucleotide sequences) shared with at least 1 other preterm neonate, as compared to term neonates (0.4%, 208/48,581), young children (0.03%, 14/46,000), and adult (0.01%, 5/48,913).

Based on the finding of increased sharing at the CDR3 nucleotide level, we hypothesized that preterm neonates would have significantly higher sharing at the amino acid level. This was confirmed in the preterm neonates, with 7.7% of their repertoire being shared with at least one other premature infant (Figure 4B). There was a stepwise regression across the life span, with term neonates sharing 3.0% of their repertoire and young children and adults sharing 1.3 and 1.2%, respectively, of their repertoire with at least one more individual (Figure 4A). There is an even greater difference among the age groups when the clones that are shared with at least 75% of the individuals within each group are compared. Preterm infants share 1.6% (601/38,145) of their repertoire, as compared to 0.18% (79/43,883) in the term infants, 0.03% (12/43,085) of young children, and 0.02% (9/45,938) of adults (Figure 4C). Remarkably, 0.18% (69/38,145) of the premature neonatal naïve CD8^+^ TCR repertoire was present within 100% of the individuals tested, and this dropped to 0.04% (18/43,883) for clones shared between all term neonates. Children and adults had very low TCR sequences shared between all individuals (0.002% for both, 1/43,085 and 1/45,938 for children and adults, respectively) (Figure 4D).

We next examined the dominance of the public TCR clones (CDR3 amino acid sequences) shared among all four preterm neonates. The 10 most frequent clones for each individual were determined, and then queried for the presence of clones common to all of the samples within that particular age group. On average, 3 of the 10 most frequent preterm clones (30%) were shared among all preterm neonates, indicating that these broadly shared clones dominate the repertoire. In contrast, 2 of the 10 most frequent term clones (20%) were shared among all term neonates. Finally, when the 10 most frequent clones present in each individual young child or adult were examined, none of these were shared. Thus, not only are public TCR clones found more frequently in preterm neonates, but they also are dominant clones in their repertoire.

### There Is Dominant TRBV Usage in the Public Repertoire

We analyzed the TRBV family usage of the preterm and term highly shared clones (shared among all 4 preterm samples and 4–5 term samples) to see if there was preferential TRBV family usage for these public clones. Overall, the distribution of V family usage in both the preterm and term neonates is similarly skewed, with the majority of public preterm clones using TRBV05, TRBV07, TRBV12, and TRBV27, and the public shared term clones using TRBV05, TRBV07, TRBV09, and TRBV27 (Figure 4E).

### The Preterm Public Repertoire Exhibits Convergent Recombination

Convergent recombination describes the process whereby multiple recombination events “converge” to produce the same nucleotide sequence and multiple nucleotide sequences converge to encode the same amino acid sequence (28). This process enables some TCR sequences to be produced at the amino acid sequence more frequently than others. TCRβ sequences with convergent features are present at higher copy numbers and shared between individuals (28). We first queried the repertoires to determine if there was evidence of convergent recombination in the total repertoire within each individual sample. In the preterm samples, there were on average 1.16 nucleotide CDR3 sequences for each amino acid CDR3 sequence, 1.07 in the term neonates, and 1.05 in the young children and adults (*p* < 0.05 for each comparison of preterm to each of the other age groups). Thus, the overall repertoire of the preterm exhibited a higher degree of convergence. Therefore, we questioned whether convergent recombination would be present among the public highly shared clones of the preterm repertoire. Upon analysis of the most highly shared clones (common to all samples/age group), the preterm neonate utilized 13.2 nucleotide rearrangements per highly shared amino acid CDR3 sequence. Thus, 913 single TCR nucleotide sequences in the preterm neonates give rise to just 69 highly shared TCR amino acid CDR3 sequences found in all preterm neonates. In comparison, the 18 TCR amino acid CDR3 sequences shared within all term neonates were generated by 176 single TCR nucleotide sequences, and therefore term neonates utilized 9.7 nucleotide rearrangements per shared amino acid CDR3 (*p* < 0.05, Figure 4F). Thus, the public shared sequences of the preterm neonate are more convergent than the term neonates.

### The Preterm Public Repertoire Persists across the Life Span

**Figure 5:**
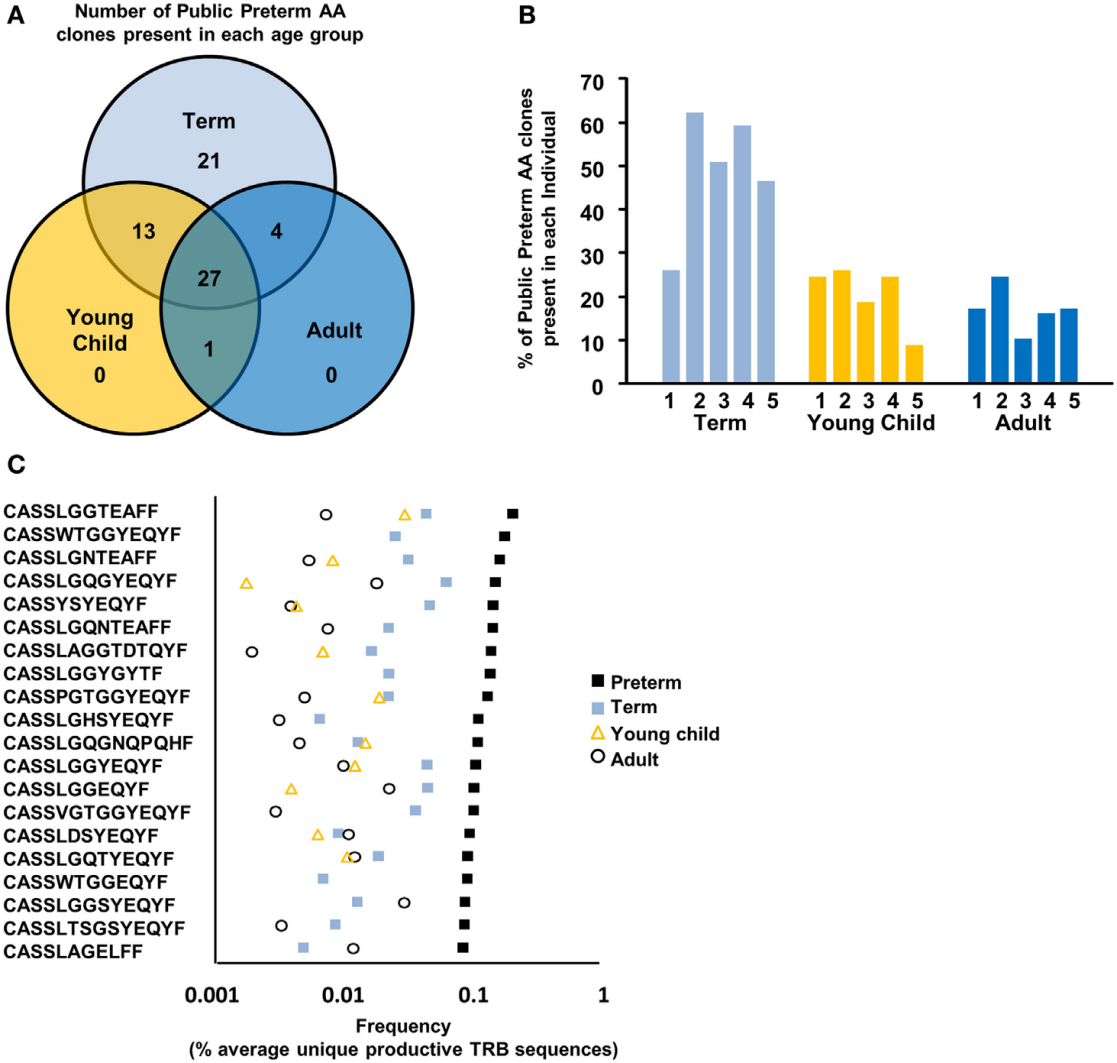
The preterm public repertoire persists across the life span. **(A)** The repertoires of the older age groups were queried for the presence of the 69 clones shared among all 4 preterm individuals. **(B)** The percentage of the 69 highly public preterm clones in each older individual is presented. **(C)** The repertoires of the older age groups were queried for the presence of the 20 most frequent highly shared preterm clones.

We next investigated whether the highly shared clones in the preterm repertoire were unique to the preterm neonate, or if they persisted into adulthood. Surprisingly, clones which were highly shared among the preterm infants (present in all four of the preterm samples) were found to persist across the life span (Figure 5A). Each individual’s repertoire was queried for the 69 highly shared preterm clones (Figure 5B). On average, each term neonate had 34/69 highly shared clones (49%); this dropped with age, with young children having 14/69 clones (20%) and adults having 12/69 clones (17%) (Figure 5B). We also examined the clones which were shared among 3 preterms (532 clones) and found that, on average, each term neonate had 147/532 clones (27.6%), young children had 56/532 (10.6%), and adults had 54/532 (10.2%).

To evaluate the frequency over the life span of individual public preterm clones, we determined the 20 most prevalent clones shared among four preterm neonates (Figure 4D) and evaluated the frequency of these public clones in the older age groups (Figure 5C). Eleven of the 20 most prevalent, public, preterm clones were present in all four age groups. This indicates these prevalent, public preterm clones persist, but their frequency decreases over the life span. Together, these data indicate that the most prevalent, public preterm clones remain public throughout life, albeit their frequency is reduced with age.

### The Preterm Public Repertoire Is Closer to Germline

**Figure 6:**
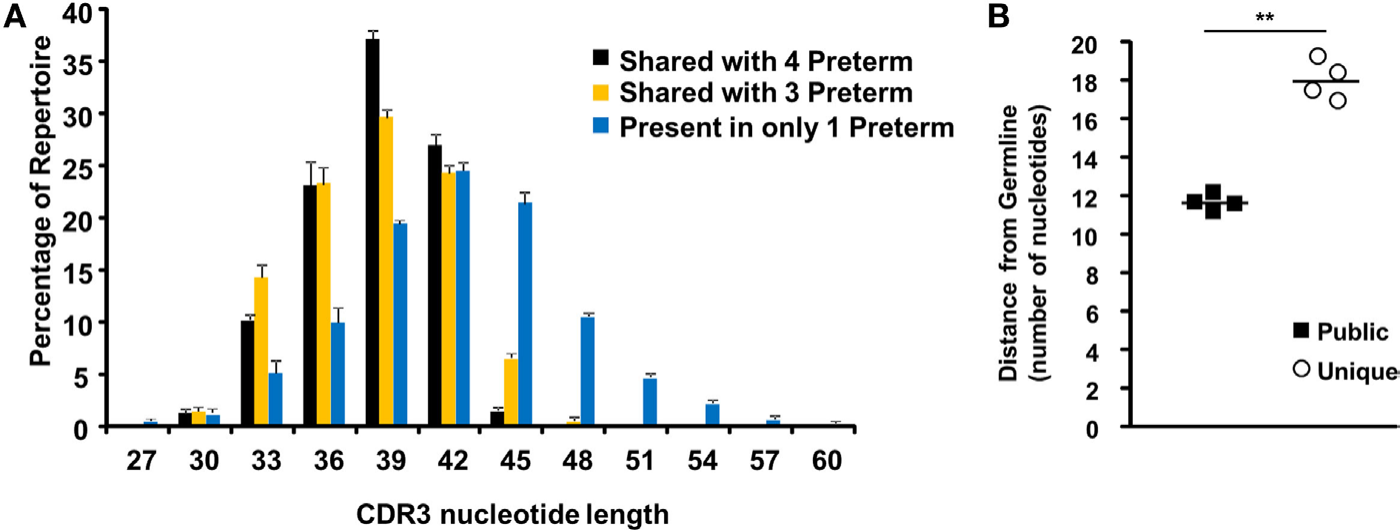
The preterm public repertoire is closer to germline. **(A)** The complementarity-determining region 3 (CDR3) nucleotide length frequency (mean ± SEM) for all unique productive sequences within each of the specified set of clones in the preterm individuals is shown. **(B)** Distance from germline is calculated based on number of nucleotides inserted or trimmed at the gene junctions within the highly public repertoire and the repertoire unique to each of the preterm neonates. Unpaired Student’s *T*-test was performed. ** represents *p* < 0.01.

Previously, it has been demonstrated that public clones have shorter CDR3 lengths (29); therefore, we determined the CDR3 lengths for the 69 amino acid clones shared among 4 preterm neonates, the 532 amino acid clones shared among 3 preterm neonates, and the 35,201 amino acid clones present in only one preterm sample (Figure 6A). The nucleotide CDR3 lengths of the clones present in one preterm individual mirrors the CDR3 length of the total preterm repertoire (Figures 3A and 6A), as these clones dominate the total repertoire. However, the highly public clones demonstrate a shift in the CDR3 nucleotide length, with a peak at 39, as opposed to the peak of 42 in the unique repertoire, and notably no clones with a nucleotide length greater than 45.

Public clones tend to have less N1 and N2 insertions and junctional trimming; as a result, these clones are closer to the germline sequence (30). Therefore, we next determined the distance to germline as described above. Indeed, those clones which are public (shared among all preterm individuals) are closer to germline as compared to clones unique to one preterm individual (Figure 6B). Unique clones within the repertoire were the most diverse and the furthest from germline. Thus, the public shared clones found in the preterm neonates largely stem from the fact that they are more germline gene encoded while the very large pool of unique clones in their repertoire are less germline in sequence.

## Discussion

Using cord blood samples from extremely preterm and term neonates, we have been able to demonstrate significant changes which occur in the naïve CD8^+^ TCR repertoire over the final trimester of pregnancy. Analysis of the evolution of the T cell repertoire may provide fundamental insights into maturation of immunocompetence and may help define risks of prematurely born neonates to infections. We find that extremely preterm neonates have shorter CDR3 lengths, with a stepwise increase in length with age; this shift in CDR3 length is due to fewer N1 and N2 nucleotide additions in preterm compared to term neonates. These N nucleotides are added by the enzyme TdT (31), suggesting that this activity matures between preterm and term neonates and continues to increase over the first year of life, consistent with previous work (32). We also report less junctional trimming in the extremely preterm neonate, as compared to term neonates and young children, which contributes to the generation of more germline CDR3 regions in preterm neonates.

Although there has previously been investigation of the B cell repertoire development (17, 33–35), little has been done to elucidate the T cell repertoire, particularly that of the preterm neonate. Rechavi et al. examined total CD4^+^ and CD8^+^ TCR repertoire by NGS in bulk T cells from four fetuses ranging from 12 to 26 gestational weeks age and compared them to three children aged 9 and 48 months old (18). They reported that fetuses had fewer nucleotide additions and less trimming compared to children. Our study examined viable, extremely preterm neonates, as opposed to elective terminations, and compared them to term neonates. We focused on the TCR repertoire of CD8^+^ T cells given their critical role in viral immunity. We find that the preterm neonates are born with naïve CD8^+^ TCR repertoires which are closer to germline, and therefore reveal important naïve CD8^+^ TCR repertoire changes over the last trimester of pregnancy.

Preterm neonates had a naïve CD8^+^ TCR repertoire with very little clonal expansion compared to the older age groups. This may suggest that the stochastic generation of TCR sequences in the thymus of preterm neonates is subject to different constraints of HLA affinity, thus allowing lower affinity clones into the periphery. These lower affinity clones are potentially less likely to undergo homeostatic proliferation (26, 36, 37). Indeed, when we examined 3-day-old mice, we have found that they utilize lower affinity TCR in their CTL response against influenza virus and that this rapidly changes over the first week of life when new higher affinity TCR clones are generated and recruited to the CTL response (9). These 3-day-old neonates have a much less diverse repertoire, as measured by the iChao1 index. The iChao1 of the 3-day-old murine naïve CD8^+^ T cells is 10-fold lower in richness than 7-day-old neonates and a 100-fold lower than murine adults. Similarly, the human preterm neonate also has a reduction in richness of their repertoire as estimated by the iChao1 index. Many important theories in community ecology make quantitative predictions about species numbers (27). These theories can be applied to the TCR repertoire, where individual species are defined as the unique templates or T cells. The most successful methods have been non-parametric estimators which use rare frequency counts to estimate the frequency of the missing species (38, 39). The basic idea is that frequent, and therefore easily detected, templates indicate very little about the number of undetected templates. However, rare TCR templates, those which are only seen once or twice in the repertoire, can inform about the number of the undetected templates. It is extremely difficult to obtain an accurate estimate of the true total number of unique templates in a human TCR repertoire. Therefore, we have many undetected TCR templates in each of our individual samples. To circumvent this problem, an accurate lower bound for species richness is often more practical than an imprecise estimate of the total number of templates within the individual. Based on the concept that rare templates carry the most information about the number of undetected templates, the iChao1 estimator uses only the numbers of those templates seen once or twice in the repertoire to obtain the lower bound for the richness of the individual’s repertoire (27). Using this approach, we estimate that the overall richness of the preterm TCR repertoire of naïve CD8^+^ T cells is lower and encodes fewer unique TCR templates. Therefore, like the mouse, a flat, simplified naïve CD8^+^ TCR repertoire in human preterm neonates may reflect a repertoire which cannot mount high-affinity responses to antigens *in utero*, something beneficial to the fetus, but upon premature birth could also impart a defective CTL response to pathogens.

Preterm neonates had high-frequency, public clones. Others have reported a high degree of T cell clonotype sharing among healthy adults in their total T cell repertoire (30). Therefore, this degree of sharing indicates that there is either a common *in utero* antigen driven repertoire selection, or there is a developmentally determined “beginner” repertoire, which is shaped by germline sequences. These public preterm clonotypes exhibited convergent recombination, where multiple CDR3 nucleotide sequences result in few amino acid CDR3 sequences. Convergent recombination is more likely when particular TCR amino acid sequences can be encoded by nucleotide sequences that are efficiently produced or encoded by multiple available nucleotide sequences (29, 40). This is largely determined by codon degeneracy of specific amino acids in the CDR3 sequence. Certain amino acids can be encoded by several codons; two of the most degenerate amino acids, leucine and serine, were enriched in the public preterm amino acid sequences. It has been suggested that sequences are not uniformly probable, as opposed to their being non-random selection from all possible sequences (41). The small effective size of the CTL CDR3 sequence repertoire is primarily attributable to the fact that CDR3 sequences with large numbers of junctional insertions have very low probability (41). Sequences with few junctional insertions are more probable than those with many insertions and consequently are more likely to be observed in multiple individuals.

We found that public clonotypes from preterm neonates were most frequent in the preterm neonates, but also persisted into adulthood, consistent with previous work (15). There is significant overlap of adult TCR repertoires, independent of HLA sharing or racial background (41). Of note, our finding of 1% sharing among any two of the adult samples is proportionally consistent with previous work which did deep sequencing of the adult naïve TCR repertoire (41). Public clones could be persisting across the life span for two reasons: public clones could be replenished by thymic output or these clones undergo homeostatic proliferation and are long-lived. Although thymic output decreases substantially across the life span, there is some thymic output which continues to occur. Future work could address this question by comparing the recent thymic emigrant repertoire to the rest of the adult naïve repertoire.

In our study, the preterm neonatal public sequences had fewer non-templated insertions and junctional trimming, resulting in shorter and more germline CDR3 regions. This raises the question whether these clonotypes can provide protection. Components of the CTL response to Epstein–Barr virus are characterized by highly conserved TCRβ CDR3 sequences that are found in multiple individuals and are also encoded by nucleotide sequences with few junctional insertions (28, 40). Such TCRβ clonotypes which are shared between individuals are thought to play an important role in the efficacy of pathogen-specific responses and control of infection (42). There is a high degree of overlap between the memory and naïve repertoires within individuals, and a substantial portion of those clonotypes found in both the naïve and memory pool consist of TCRβ clonotypes that are shared between individuals. These shared TCRβ clonotypes are present at a higher frequency in the memory and naïve repertoires, as opposed to unique clonotypes (29). Thus, the generation of public clonotypes in the preterm neonatal repertoire may offer a degree of protection, although this may not be as efficient as higher affinity TCR responses mounted by term neonates.

There is evidence of increased TCR cross-reactivity in TdT-deficient cells, and greater cross-reactivity has been associated with higher self-pMHC reactivity (43, 44). Therefore, the public, germline clonotypes in the preterm neonate may be the most cross-reactive. The existence of public clonotypes in antiviral CTL responses suggests the possibility that the Vβ, Dβ, and Jβ segment sequences that contribute to recurrently generated TCRs could be subjected to evolutionary pressures favoring sequences recognizing antigens from common pathogens, because these sequences are present in the germline. The presence of public clones at high frequencies in preterm neonates is perhaps the initial line of defense with which nature arms the fetus to mount a broadly protecting CTL response. However, these cross-reactive, germline clonotypes could be pathogenic if left unchecked. An imbalance in effector T cells and Tregs has been postulated as a root cause of a potentially lethal condition in the premature infant, necrotizing enterocolitis (45). If there are broad, evolutionary pressures which lead to a public CD8^+^ T cell repertoire, it is not unreasonable to speculate that conventional CD4^+^ T cells and Tregs have a similar public repertoire. Further investigations into the CD4^+^ TCR repertoire are ongoing in our laboratory.

Limitations of our study include the small sample size and variation in productive templates in the preterm neonates. Small sample size and sequencing variation are frequent limitations of neonatal TCR repertoire work (15, 17, 18), particularly viable preterm neonates. Delayed cord clamping is practiced now routinely based on work showing decreased need for transfusion and improved circulatory stability (46), which leads to decreased blood volume for TCR repertoire analysis. In addition to conducting studies with more patients, prospective, longitudinal studies of both preterm and term neonates need to be completed to track T cell development within individuals born at different gestational ages.

Here, after a comprehensive evaluation of the development of the naïve CD8^+^ TCR repertoire in the human, we demonstrated a less rich repertoire, characterized by public, convergent rearrangements in the extremely preterm neonate. This public repertoire decreases with age and persists into adulthood. We report here that the preterm neonatal naïve CD8^+^ TCR repertoire significantly differs from that of the term neonate; this repertoire immaturity potentially contributes to their increased infection susceptibility. Understanding in greater depth the TCR repertoire of preterm neonates provides better insight into the underlying mechanisms of the increased morbidity and mortality of this sensitive population.

## Ethics Statement

This study was approved by the Institutional Review Board of Drexel University College of Medicine and Erasmus Medical Center. Those patients with fetal demise, maternal active HSV infection, and mothers with known HIV infection were excluded. There was a waiver of documentation of informed consent for cord blood collection because of the minimal risk. For peripheral blood collection from children and adults, written informed consent was obtained. Subjects gave written informed consent in accordance with the Declaration of Helsinki.

## Author Contributions

Concept and experimental design were conceived by AC and PK; experiments were performed by AC, JH, YM, AF, and OK; data curation was overseen by AC; data analysis was done by AC, DZ, ES, MB, and PK. All authors reviewed manuscript. Manuscript was written by AC and PK.

## Conflict of Interest Statement

The authors declare that the research was conducted in the absence of any commercial or financial relationships that could be construed as a potential conflict of interest.

## References

[1] Stoll BJ Hansen NI Bell EF Shankaran S Laptook AR Walsh MC Neonatal outcomes of extremely preterm infants from the NICHD neonatal research network. Pediatrics (2010) 126(3):443–56. 10.1542/peds.2009-2959 20732945 PMC2982806

[2] Patel RM Kandefer S Walsh MC Bell EF Carlo WA Laptook AR Causes and timing of death in extremely premature infants from 2000 through 2011. N Engl J Med (2015) 372(4):331–40. 10.1056/NEJMoa1403489 25607427 PMC4349362

[3] Berrington JE Hearn RI Bythell M Wright C Embleton ND Deaths in preterm infants: changing pathology over 2 decades. J Pediatr (2012) 160(1):49–53.e1. 10.1016/j.jpeds.2011.06.046 21868028

[4] Williams EJ Embleton ND Clark JE Bythell M Ward Platt MP Berrington JE. Viral infections: contributions to late fetal death, stillbirth, and infant death. J Pediatr (2013) 163(2):424–8. 10.1016/j.jpeds.2013.02.004 23507026

[5] Kagi D Ledermann B Burki K Hengartner H Zinkernagel RM CD8+ T cell-mediated protection against an intracellular bacterium by perforin-dependent cytotoxicity. Eur J Immunol (1994) 24(12):3068–72. 10.1002/eji.1830241223 7805735

[6] Bender BS Croghan T Zhang L Small PA Jr. Transgenic mice lacking class I major histocompatibility complex-restricted T cells have delayed viral clearance and increased mortality after influenza virus challenge. J Exp Med (1992) 175(4):1143–5. 10.1084/jem.175.4.1143 1552285 PMC2119177

[7] Fernandez MA Evans IA Hassan EH Carbone FR Jones CA. Neonatal CD8^+^ T cells are slow to develop into lytic effectors after HSV infection in vivo. Eur J Immunol (2008) 38(1):102–13. 10.1002/eji.200636945 18081035

[8] Adkins B. T-cell function in newborn mice and humans. Immunol Today (1999) 20(7):330–5. 10.1016/S0167-5699(99)01473-5 10379052

[9] Carey AJ Gracias DT Thayer JL Boesteanu AC Kumova OK Mueller YM Rapid evolution of the CD8+ TCR repertoire in neonatal mice. J Immunol (2016) 196(6):2602–13. 10.4049/jimmunol.1502126 26873987 PMC4779665

[10] Lines JL Hoskins S Hollifield M Cauley LS Garvy BA. The migration of T cells in response to influenza virus is altered in neonatal mice. J Immunol (2010) 185(5):2980–8. 10.4049/jimmunol.0903075 20656925 PMC2924920

[11] Goncalves P Ferrarini M Molina-Paris C Lythe G Vasseur F Lim A A new mechanism shapes the naive CD8+ T cell repertoire: the selection for full diversity. Mol Immunol (2017) 85:66–80. 10.1016/j.molimm.2017.01.026 28212502

[12] Holt PG Jones CA The development of the immune system during pregnancy and early life. Allergy (2000) 55(8):688–97. 10.1034/j.1398-9995.2000.00118.x 10955693

[13] Raaphorst FM Kaijzel EL van Tol MJ Vossen JM van den Elsen PJ. Non-random employment of V beta 6 and J beta gene elements and conserved amino acid usage profiles in CDR3 regions of human fetal and adult TCR beta chain rearrangements. Int Immunol (1994) 6(1):1–9. 10.1093/intimm/6.1.1 8148317

[14] Schelonka RL Raaphorst FM Infante D Kraig E Teale JM Infante AJ. T cell receptor repertoire diversity and clonal expansion in human neonates. Pediatr Res (1998) 43(3):396–402. 10.1203/00006450-199803000-00015 9505280

[15] Britanova OV Shugay M Merzlyak EM Staroverov DB Putintseva EV Turchaninova MA Dynamics of individual T cell repertoires: from cord blood to centenarians. J Immunol (2016) 196(12):5005–13. 10.4049/jimmunol.1600005 27183615

[16] Pogorelyy MV Elhanati Y Marcou Q Sycheva AL Komech EA Nazarov VI Persisting fetal clonotypes influence the structure and overlap of adult human T cell receptor repertoires. PLoS Comput Biol (2017) 13(7):e1005572. 10.1371/journal.pcbi.1005572 28683116 PMC5500008

[17] Zemlin M Hoersch G Zemlin C Pohl-Schickinger A Hummel M Berek C The postnatal maturation of the immunoglobulin heavy chain IgG repertoire in human preterm neonates is slower than in term neonates. J Immunol (2007) 178(2):1180–8. 10.4049/jimmunol.178.2.1180 17202383

[18] Rechavi E Lev A Lee YN Simon AJ Yinon Y Lipitz S Timely and spatially regulated maturation of B and T cell repertoire during human fetal development. Sci Transl Med (2015) 7(276):276ra25 10.1126/scitranslmed.aaa0072 25717098

[19] Melville JM Moss TJ. The immune consequences of preterm birth. Front Neurosci (2013) 7:79. 10.3389/fnins.2013.00079 23734091 PMC3659282

[20] Fike AJ Nguyen LT Kumova OK Carey AJ Characterization of CD31 expression on murine and human neonatal T lymphocytes during development and activation. Pediatr Res (2017) 82(1):133–40. 10.1038/pr.2017.81 28355204 PMC5509503

[21] Harris PA Taylor R Thielke R Payne J Gonzalez N Conde JG Research electronic data capture (REDCap) – a metadata-driven methodology and workflow process for providing translational research informatics support. J Biomed Inform (2009) 42(2):377–81. 10.1016/j.jbi.2008.08.010 18929686 PMC2700030

[22] Krzywinski M Schein J Birol I Connors J Gascoyne R Horsman D Circos: an information aesthetic for comparative genomics. Genome Res (2009) 19(9):1639–45. 10.1101/gr.092759.109 19541911 PMC2752132

[23] Pielou EC The measurement of diversity in different types of biological collections. J Theor Biol (1966) 13:131–44. 10.1016/0022-5193(66)90013-0

[24] Chiu CH Wang YT Walther BA Chao A. An improved nonparametric lower bound of species richness via a modified good-turing frequency formula. Biometrics (2014) 70(3):671–82. 10.1111/biom.12200 24945937

[25] Zemlin M Schelonka RL Bauer K Schroeder HW Jr Regulation and chance in the ontogeny of B and T cell antigen receptor repertoires. Immunol Res (2002) 26(1–3):265–78. 10.1385/IR:26:1-3:265 12403364

[26] den Braber I Mugwagwa T Vrisekoop N Westera L Mogling R de Boer AB Maintenance of peripheral naive T cells is sustained by thymus output in mice but not humans. Immunity (2012) 36(2):288–97. 10.1016/j.immuni.2012.02.006 22365666

[27] Gotelli NJ Chao A Measuring and estimating species richness, species diversity, and biotic similarity from sampling data. In: Levin SA, editor. Encyclopedia of Biodiversity. Waltham, MA: Academic Press (2013). p. 195–211.

[28] Venturi V Price DA Douek DC Davenport MP. The molecular basis for public T-cell responses? Nat Rev Immunol (2008) 8(3):231–8. 10.1038/nri2260 18301425

[29] Venturi V Quigley MF Greenaway HY Ng PC Ende ZS McIntosh T A mechanism for TCR sharing between T cell subsets and individuals revealed by pyrosequencing. J Immunol (2011) 186(7):4285–94. 10.4049/jimmunol.1003898 21383244

[30] Hou X Wang M Lu C Xie Q Cui G Chen J Analysis of the repertoire features of TCR beta chain CDR3 in human by high-throughput sequencing. Cell Physiol Biochem (2016) 39(2):651–67. 10.1159/000445656 27442436

[31] Desiderio SV Yancopoulos GD Paskind M Thomas E Boss MA Landau N Insertion of N regions into heavy-chain genes is correlated with expression of terminal deoxytransferase in B cells. Nature (1984) 311(5988):752–5. 10.1038/311752a0 6092963

[32] Feeney AJ. Junctional sequences of fetal T cell receptor beta chains have few N regions. J Exp Med (1991) 174(1):115–24. 10.1084/jem.174.1.115 1711558 PMC2118868

[33] Schroeder HW Jr Wang JY. Preferential utilization of conserved immunoglobulin heavy chain variable gene segments during human fetal life. Proc Natl Acad Sci U S A (1990) 87(16):6146–50. 10.1073/pnas.87.16.6146 2117273 PMC54489

[34] Pascual V Verkruyse L Casey ML Capra JD Analysis of Ig H chain gene segment utilization in human fetal liver. Revisiting the “proximal utilization hypothesis”. J Immunol (1993) 151(8):4164–72. 8409393

[35] Baskin B Islam KB Smith CI. Characterization of the CDR3 region of rearranged alpha heavy chain genes in human fetal liver. Clin Exp Immunol (1998) 112(1):44–7. 10.1046/j.1365-2249.1998.00547.x 9566788 PMC1904953

[36] Silva SL Sousa AE Establishment and maintenance of the human naive CD4+ T-cell compartment. Front Pediatr (2016) 4:119 10.3389/fped.2016.00119 27843891 PMC5086629

[37] Pekalski ML Ferreira RC Coulson RM Cutler AJ Guo H Smyth DJ Postthymic expansion in human CD4 naive T cells defined by expression of functional high-affinity IL-2 receptors. J Immunol (2013) 190(6):2554–66. 10.4049/jimmunol.1202914 23418630 PMC3614027

[38] Colwell RK Coddington JA. Estimating terrestrial biodiversity through extrapolation. Philos Trans R Soc Lond B Biol Sci (1994) 345(1311):101–18. 10.1098/rstb.1994.0091 7972351

[39] Chao A Non-parametric estimation of the number of classes in a population. Scand J Stat (1984) 11:265–70.

[40] Venturi V Chin HY Asher TE Ladell K Scheinberg P Bornstein E TCR beta-chain sharing in human CD8+ T cell responses to cytomegalovirus and EBV. J Immunol (2008) 181(11):7853–62. 10.4049/jimmunol.181.11.7853 19017975

[41] Robins HS Srivastava SK Campregher PV Turtle CJ Andriesen J Riddell SR Overlap and effective size of the human CD8+ T cell receptor repertoire. Sci Transl Med (2010) 2(47):47ra64 10.1126/scitranslmed.3001442 PMC3212437 20811043

[42] Price DA Asher TE Wilson NA Nason MC Brenchley JM Metzler IS Public clonotype usage identifies protective Gag-specific CD8+ T cell responses in SIV infection. J Exp Med (2009) 206(4):923–36. 10.1084/jem.20081127 19349463 PMC2715115

[43] Gavin MA Bevan MJ. Increased peptide promiscuity provides a rationale for the lack of N regions in the neonatal T cell repertoire. Immunity (1995) 3(6):793–800. 10.1016/1074-7613(95)90068-3 8777724

[44] Mandl JN Monteiro JP Vrisekoop N Germain RN T cell-positive selection uses self-ligand binding strength to optimize repertoire recognition of foreign antigens. Immunity (2013) 38(2):263–74. 10.1016/j.immuni.2012.09.011 23290521 PMC3785078

[45] Weitkamp JH Koyama T Rock MT Correa H Goettel JA Matta P Necrotising enterocolitis is characterised by disrupted immune regulation and diminished mucosal regulatory (FOXP3)/effector (CD4, CD8) T cell ratios. Gut (2013) 62(1):73–82. 10.1136/gutjnl-2011-301551 22267598 PMC3606820

[46] Rabe H Diaz-Rossello JL Duley L Dowswell T Effect of timing of umbilical cord clamping and other strategies to influence placental transfusion at preterm birth on maternal and infant outcomes. Cochrane Database Syst Rev (2012) 8:CD003248 10.1002/14651858.CD003248.pub3 22895933

