# Supplementary material for: Public Clonotypes and Convergent Recombination Characterize the Naïve CD8+ T-Cell Receptor Repertoire of Extremely Preterm Neonates
Source: Front Immunol. 2017 Dec 19;8:1859. doi: 10.3389/fimmu.2017.01859 (PMC5742125; doi:10.3389/fimmu.2017.01859)
Supplement: Supplementary file 1 [file Table_1.docx]

**Supplementary table 1**

Clinical Information about the preterm samples

| **Sample Code** | **Sex** | **Gestational Age** | **Birth weight (g)** | **Birth weight Percentile** | **Indication for Delivery** | **Doses of Betamethasone** |
| --- | --- | --- | --- | --- | --- | --- |
| Preterm_23_3 | Male | 23 weeks  3 days | 640 | 50-75% | Termination of pregnancy for craniosynostosis, no other congenital anomalies | 0 |
| Preterm_25_5 | Female | 25 weeks  5 days | 1140 | 95% | Induced for severe preclampsia, failed control of hypertension with Magnesium Sulfate | 2 |
| Preterm_26_B_2 | Male | 26 weeks  2 days | 1040 | 50-75% | Spontaneous, failed tocolysis with Nifedipine. Twin gestation. | 1 |
| Preterm_27_4 | Female | 27 weeks  4 days | 1400 | 95% | Spontaneous, failed tocolysis with Nifedipine. | 2 |
