# Supplementary material for: Public Clonotypes and Convergent Recombination Characterize the Naïve CD8+ T-Cell Receptor Repertoire of Extremely Preterm Neonates
Source: Front Immunol. 2017 Dec 19;8:1859. doi: 10.3389/fimmu.2017.01859 (PMC5742125; doi:10.3389/fimmu.2017.01859)
Supplement: Supplementary file 2 [file Table_2.docx]

**Supplementary table 2**

Summary of the number of sequences that were used to analyze the TRB repertoire

| **Sample Code** | **Total Templates** | **Prod Templates** | **Total rearrangements** | **Productive Nucleotide sequences** | **Productive AA sequences** | **Productive clonality (Pielou’s evenness)** |
| --- | --- | --- | --- | --- | --- | --- |
| Preterm_23_3 | 46097 | 33085 | 39962 | 27927 | 22079 | 0.0115 |
| Preterm_25_5 | 1738 | 1191 | 1718 | 1177 | 1090 | 0.0007 |
| Preterm_26_B_2 | 8668 | 6260 | 8373 | 6022 | 5376 | 0.002 |
| Preterm_27_4 | 23400 | 17021 | 22133 | 15959 | 13217 | 0.0038 |
|  |  |  |  |  |  |  |
| Cord_1_NWK206 | 11052 | 8136 | 10946 | 8009 | 7659 | 0.0236 |
| Cord_2_NWK211 | 16590 | 12682 | 15535 | 11778 | 10951 | 0.032 |
| Cord_3_NWK213 | 16072 | 12179 | 15973 | 11912 | 11058 | 0.0243 |
| Cord_4_NWK231 | 12766 | 9591 | 12721 | 9413 | 8736 | 0.0229 |
| Cord_5_NWK247 | 10727 | 7888 | 10591 | 7707 | 7209 | 0.0277 |
|  |  |  |  |  |  |  |
| Infant_1_NWK51 | 11331 | 9391 | 11126 | 9092 | 8592 | 0.0277 |
| Infant_2_NWK134 | 11121 | 8981 | 11052 | 8822 | 8404 | 0.0221 |
| Infant_3_NWK39 | 12519 | 10149 | 12335 | 9908 | 9414 | 0.025 |
| Infant_4_NWK174 | 13746 | 11534 | 13660 | 11408 | 10774 | 0.023 |
| Infant_5_NWK31 | 8652 | 6899 | 8510 | 6787 | 6505 | 0.0264 |
|  |  |  |  |  |  |  |
| Adult_1_15-04 | 15728 | 13067 | 15373 | 12639 | 12052 | 0.0321 |
| Adult_2_15-07 | 13698 | 11229 | 13595 | 11037 | 10461 | 0.0237 |
| Adult_3_15-03 | 11032 | 9169 | 10288 | 8503 | 8127 | 0.0693 |
| Adult_4_15-05 | 11041 | 9244 | 10777 | 8962 | 8530 | 0.0298 |
| Adult_5_15-06 | 9416 | 7890 | 9345 | 7777 | 7407 | 0.0323 |
