# Supplementary figures and images for: Public Clonotypes and Convergent Recombination Characterize the Naïve CD8+ T-Cell Receptor Repertoire of Extremely Preterm Neonates
Source: Front Immunol. 2017 Dec 19;8:1859. doi: 10.3389/fimmu.2017.01859 (PMC5742125; doi:10.3389/fimmu.2017.01859)

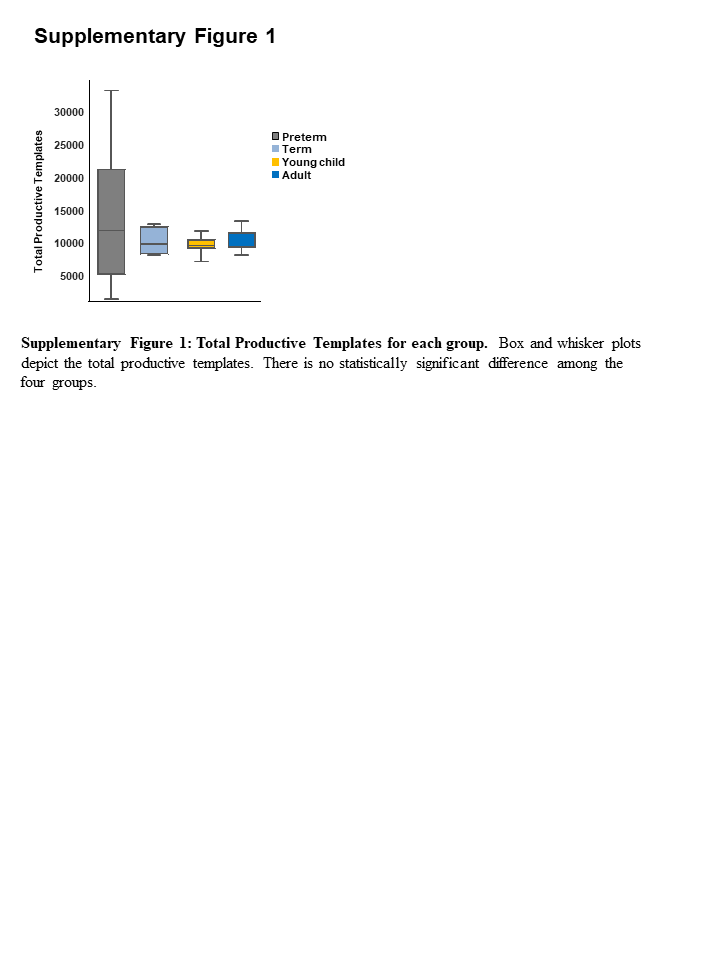

Supplement: Supplementary file 3 [file Image_1.TIF]
